# Patterns of flavored e-cigarette use among adult vapers in the USA: an online cross-sectional survey of 69,233 participants

**DOI:** 10.1186/s12954-023-00876-w

**Published:** 2023-10-14

**Authors:** Konstantinos Farsalinos, Christopher Russell, Riccardo Polosa, Konstantinos Poulas, George Lagoumintzis, Anastasia Barbouni

**Affiliations:** 1 Department of Public and Community Health, University of West Attica, Aigaleo, Greece; 2 Department of Pharmacy, University of Patras, Patras, Greece; 3 Russell Burnett Research & Consultancy Ltd, Glasgow, UK; 4 Department of Clinical and Experimental Medicine, University of Catania, Catania, Italy; 5 Center of Excellence for the Acceleration of Harm Reduction (CoEHAR), Università Di Catania, Catania, Italy; 6 Centre for the Prevention and Treatment of Tobacco Addiction (CPCT), Teaching Hospital “Policlinico - V. Emanuele,” University of Catania, Catania, Italy; 7 Institute of Internal Medicine, Teaching Hospital “AOU Policlinico - V. Emanuele – S. Marco,” University of Catania, Catania, Italy

## Abstract

**Background:**

Flavored e-cigarettes remain a controversial topic with regulators planning or already implementing restrictions worldwide. In this study, we examined patterns of flavor use in e-cigarettes among a convenience sample of US adult vapers.

**Methods:**

Participants aged ≥ 18 years who reported ever using an e-cigarette were included in the study (*N* = 69,233) and responded to an online questionnaire. Their smoking status was recorded as well as patterns of flavor use at e-cigarette use initiation, at the time of the survey and at the time of smoking cessation (for participants who used to smoke and were using e-cigarettes at the time of quitting).

**Results:**

The most popular flavors at e-cigarette use initiation were fruit (82.8%), followed by dessert/pastry/bakery (68.6%) and candy/chocolate/sweet (52.2%). Slightly higher prevalence of using fruit and dessert/pastry/bakery flavors was observed in those who never smoked compared to those who were currently and formerly smoking. Tobacco flavors were used by 20.8% of the participants and was by far the least prevalent among participants who never smoked. Similar patterns were observed with participants’ choices at the time of the survey, but tobacco flavor use was substantially reduced (7.7%). Only 2.1% reported tobacco as the single most often used flavor. The most prevalent flavor at the time of quitting smoking was again fruit (83.3%), followed by dessert/pastry/bakery (68.0%) and candy/chocolate/sweet (44.5%). These flavors were considered the most helpful for quitting smoking. Tobacco flavor use at the time of smoking cessation was reported by 15.0%, while 9.3% considered it helpful for quitting smoking.

**Conclusion:**

Non-tobacco flavors were popular among the US adult vapers who participated in the study, and were popular choices at the time of quitting smoking for those who formerly smoked. Tobacco flavor use prevalence was low and was further reduced over time. Regulators should consider the flavor choice of adult consumers, especially those who quit smoking, when preparing legislation on flavored e-cigarettes.

**Supplementary Information:**

The online version contains supplementary material available at 10.1186/s12954-023-00876-w.

## Background

Electronic cigarettes (e-cigarettes) have been marketed in recent years as alternative to smoking products and have rapidly grown in popularity in several countries including the USA [1–3]. Nationally representative population surveys suggest that they are the most popular smoking cessation aid in the USA [4, 5]. E-cigarettes consist mainly of a battery and an atomizer where liquid is stored and gets evaporated by energy supplied to an electrical resistance. The liquid contains mainly propylene glycol and glycerol, with the option to include nicotine. A major characteristic of the e-cigarette market is the availability of a variety of flavorings in e-liquids. Besides tobacco-like flavors, consumers can choose flavors consisting of fruits, sweets, drinks and beverages and many more. An estimated 7700 unique e-liquid flavors were identified in 2014 [6], and it is very likely that the number has further increased in recent years. Evidence from cross-sectional surveys of dedicated vapers suggests that people who smoke tend to initiate e-cigarette use with tobacco-flavored e-cigarettes but transition to exclusive or predominant use of non-tobacco-flavored products—particularly fruit, sweet, and dessert flavors—with increased frequency and duration of e-cigarette use [3, 7, 8]. Former-smoking vapers report that switching between flavors within the same day is common and that regular use of multiple e-liquid flavors was associated with significantly higher odds of having quit smoking, with fruit and sweet flavors being the most popular choices among established long-term vapers [8].

The availability of so many different flavorings has been criticized by authorities stating that there is a potential to attract youngsters. This could potentially habituate youth to the effects of nicotine and, in turn, youth who would not have smoked in the absence of flavored e-cigarettes will “graduate” to use more harmful combustible tobacco products that deliver nicotine more efficiently [9]. Studies have shown that the majority of youth and young adults who have ever tried an e-cigarette started their use with fruit or sweet flavors rather than a tobacco flavor, while rates of use of flavored tobacco products are higher among youth and young adults than among older adults [7, 10, 11]. Regulatory authorities have tried to address these concerns, mainly by considering flavors restrictions or bans. In the USA, particularly after the acute lung disease outbreak in 2019 (named EVALI), several states introduced flavors bans for e-cigarettes, despite the fact that this outbreak was most likely related to illicit to tetrahydrocannabinol oil use rather than flavored nicotine e-cigarettes [12, 13]. In Europe, there are also discussions about flavors restrictions in the upcoming Tobacco Products Directive.

Understanding the patterns of flavor use among adults is important since any overly restrictive regulatory decisions (e.g., a ban on popular flavors) could have unintended consequences among established adult vapers who may have reduced or quit smoking with the help of e-cigarettes. Risk reduction in people who smoke and quit by switching to e-cigarettes is one of the determinants of the public health impact of e-cigarettes [14]. Therefore, any regulatory framework should consider the balance between protecting population subgroups from unintended and undesired use and causing harm to people who use e-cigarettes as smoking substitutes.

The main purpose of this study was to analyze the patterns of flavored e-cigarette use in a large sample of dedicated adult vapers residing in the USA. Additionally, the study focused on comparing flavor use between current-smoking vapers (dual use) and former-smoking vapers, and on specifically examining patterns of flavor use among former-smoking vapers at the time of quitting smoking.

## Methods

### Study sample and online platform

The study sample consisted of individuals aged 18 and older living in the USA who have ever used an e-cigarette (even a single puff). Participants were invited to complete an online questionnaire that was available through Dacima Survey software (Dacima, Montreal, Quebec, Canada). Of note, this tool is FDA 21 CFR Part 11 compliant (https://www.dacimasoftware.com/healthcare/dacima-survey-a-web-survey-software-that-is-fda-21cfr-part-11-compliant/). Before entering the main survey questionnaire, participants had to read an informed consent form and check that they agreed to participate. The informed consent presented the purpose of the survey, the names and contact details of the study investigators, information about who was eligible to take part and how survey data would be used, and assurances of participant anonymity and confidentiality. Subsequently, participants were asked if they were permanent residents of the USA, their age and if they had ever used an e-cigarette (even once or twice). Participants satisfying these inclusion criteria (adults, permanent residents of the USA and having used an e-cigarette) were directed to the main questionnaire. No financial or other incentive was offered in exchange for participation. The study was approved by the ethics committee of the University of Patras in Greece.

The questionnaire was open for participation from April 3rd to May 2nd, 2018. No personal identifying details were collected, besides the usual demographic information collected in any type of cross-sectional survey (see Results section). The IP address was recorded with the sole purpose of removing double entries.

### Questionnaire design

The questionnaire assessed in detail the past and current smoking status of participants. Participants’ smoking status were defined as current smoking if they were smoking in the past 30 days. Former smoking were defined as having smoked in the past (even 1 or 2 puffs) but not smoked in the past 30 days. Never smoking was defined as having never smoked a tobacco cigarette.

All participants were by definition ever vapers. The patterns of use and reasons for e-cigarette use initiation were recorded. Specifically, the age of the participants when initiating e-cigarette use and when becoming regular and daily vapers was recorded. Additionally, participants were asked to report whether they used e-cigarettes at the time of the survey every day, some days or not at all. A specific question among participants who former smoked examined whether they were using e-cigarettes at the time of quitting smoking by asking “Thinking back to when you quit smoking cigarettes completely, were you using an e-cigarette/vaping device…,” with response options being “Every day,” “Some days,” and “Not at all.” This question was considered important to more reliably identify those who former smoked and had quit smoking with the help of e-cigarettes. Questions about e-cigarette flavor use were asked in three sections, addressing three different periods: (A) At the time of e-cigarette use initiation; (B) At the time of survey participation; and (C) At the time of quitting smoking. The latter was recorded only for participants who former smoked and responded that they were using e-cigarettes at the time of quitting. At all time points, participants were asked to report all types of flavors used regularly (multiple responses were allowed) but also the single flavor most often used. For both questions, a pre-determined list of flavor types was provided for the participants to choose from, specifically tobacco, menthol, mint/wintergreen, fruit, dessert/pastry/bakery, candy/chocolate/sweet, spice, coffee, alcohol/cocktail, non-alcoholic/non-coffee drink, unflavored, and other.

### Statistical analysis

Descriptive analysis was performed with the data presented as median and interquartile range (IQR) for continuous variables and proportions (%), number (*n*) and 95% confidence intervals (95%CI) for categorical variables. Comparisons between smoking groups were performed using cross-tabulations and chi-square tests with *z*-test to compare column proportions with Bonferroni correction. Paired comparisons in flavor use between different time points (e-cigarette use initiation and survey time points) were examined using nonparametric paired samples test (McNemar test). All analyses were performed with commercially available software (SPSS v. 22, Chicago IL, USA), and a *p* value of < 0.05 was considered statistically significant.

## Results

### Descriptive analysis for all participants

After removing double entries through the IP address, the study sample consisted of 69,233 adult vapers living in the USA. The reported residence state of participants is presented in Additional file 1: Table 1. Only 0.7% (*n* = 506) did not report their residence state (missing data). Participant demographics are presented in Table 1.

**Table 1:** Participant demographics (*n* = 69,233)

	Mean (SD)/% (*n*)
*Age*	34.6 (11.6)
*Gender*	
Male	72.4% (50,157)
Female	26.5% (18,341)
Transgender	0.5% (326)
*Marital status*	
Married	40.6% (28,077)
Never married	44.9% (31,066)
Divorced	11.2% (7733)
Separated	2.2% (1506)
Widowed	0.9% (611)
*Employment status*	
Not currently working for pay	16.9% (11,691)
Full-time working, at least 35 h/week	70.8% (48,999)
Part-time working, 15-34 h/week	9.9% (6823)
Part-time working, < 15 h/week	2.1% (1461)
*Education*	
Less than high school	0.9% (622)
Some high school, no diploma	4.0% (2784)
General Education Diploma (GED)	7.6% (5249)
High school graduate—diploma	25.3% (17,491)
Some college but no degree	32.9% (22,811)
Associate degree—occupational/vocational	9.4% (6521)
Associate degree—academic program	5.5% (3830)
Bachelor’s degree (ex: BA, AB, BS)	10.0% (6937)
Master’s degree (ex: MA, MS, MEng,Med, MSW)	2.1% (1484)
Professional school degree (ex: MD,DDS, DVM, JD)	0.5% (354)
Doctorate degree (ex: PhD, EdD)	0.3% (219)
*Currently enrolled in a degree program*	
Yes	9.6% (6663)
No	85.8% (59,398)
*Household income per 12 months*	
Less than $10,000	6.7% (4652)
$10,000 to $14,999	6.2% (4305)
$15,000 to $24,999	10.9% (7547)
$25,000 to $34,999	13.1% (9055)
$35,000 to $49,999	15.7% (10,882)
$50,000 to $74,999	18.3% (12,694)
$75,000 to $99,999	10.4% (7216)
$100,000 to $149,999	8.6% (5931)
$150,000 to $199,999	2.5% (1735)
$200,000 or more	2.0% (1361)

**Fig. 1:**
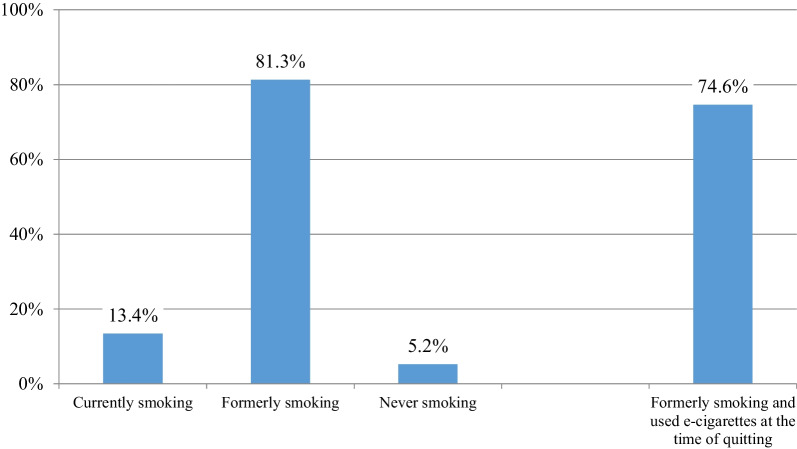
Smoking status of the study participants (*n* = 69,233)

The smoking history of participants is presented in Fig. 1. Almost 95% of participants reported having ever (currently or formerly) smoked. The majority had quit smoking, while 61% of those who were currently smoking were only smoking occasionally (on some days). Notably, 68.2% of all participants reported having quit smoking > 12 months ago, while 13.1% had quit within the past 12 months before survey participation. The median time since quitting smoking for all participants who formerly smoked was 36 months (IQR: 23–61 months). Only 5.2% of the study sample reported having never smoked. The vast majority of those who formerly smoked (91.8%, 74.6% of the study sample) reported using e-cigarettes at the time of smoking cessation. The median age of first e-cigarette use was 28 (21–37) years, while the age of initiating regular e-cigarette use was 29 (22–38) years. Almost all participants (98.9%) were using e-cigarettes in the past 30 days at the time of the survey, with most (93.5% of the whole sample) using them every day.

Table 2 presents the e-cigarette equipment and flavors used by the participants at e-cigarette use initiation. Most participants initiated e-cigarette use with advanced devices (variable voltage/wattage) or eGO-style batteries. A small minority used first-generation (cigarette-like, “ciga-like”) devices. The most popular nicotine concentration at initiation was 1–6 mg/mL followed by 18–24 mg/mL. For most participants, it was easy to find the flavors of preference at e-cigarette use initiation, which was expected considering the unrestricted large variability available at the time of the survey. Participants were asked to report all different flavors that they were using regularly, but they were also subsequently asked to report the single most regularly used flavor. The most popular flavors were fruit and dessert/pastry/bakery, while only approximately 20% reported using tobacco flavors. Similarly, fruit and dessert/pastry/bakery were the most popular single flavor used most often at e-cigarette use initiation.

**Table 2:** E-cigarette equipment and flavor use at e-cigarette use initiation (*n* = 69,233)

	%	95% CI	*N*
*First device used*			
Disposable	8.1	7.8–8.3	5635
Rechargeable ciga-like with prefilled cartridges	17.5	17.2–17.8	12,135
eGo-style	32.5	32.2–32.8	22,477
Pod mod	1.2	1.1–1.3	811
Mechanical device	3.7	3.6–3.8	2544
Variable voltage/wattage (advanced personal vaporizer)	35.6	35.2–36.0	24,657
Something else	1.4	1.3–1.5	974
*Initial nicotine concentration*			
0 mg/mL	4.1	4.0–4.2	2827
1–6 mg/mL	38.1	37.7–38.5	26,411
7–12 mg/mL	17.5	17.2–17.8	12,117
13–17 mg/mL	4.5	4.3–4.7	3083
18–24 mg/mL	26.1	25.8–26.4	18,088
25–49 mg/mL	3.3	3.2–3.4	2294
50 mg/mL or more	0.6	0.5–0.7	402
*How difficult was it to find the flavor you likes at e-cigarette use initiation?*			
Very difficult	3.1	3.0–3.2	2133
Difficult	9.9	9.7–10.1	6836
Neither easy nor difficult	21.8	21.5–22.1	15,078
Easy	27.1	26.8–27.4	18,742
Very easy	38.2	37.8–38.6	26,444
Initial flavor choice (used regularly)*			
Tobacco	20.8	20.5–21.1	14,373
Menthol	21.9	21.6–22.2	15,133
Mint/wintergreen	13.8	13.5–14.1	9581
Fruit	82.8	82.5–83.1	57,320
Dessert/pastry/bakery	68.6	68.3–68.9	47,509
Candy/chocolate/sweet	52.2	51.8–52.6	36,160
Spice	12.5	12.2–12.7	8659
Coffee	26.4	26.1–26.7	18,306
Alcohol/cocktail	7.5	7.3–7.7	5211
Non-alcoholic/non-coffee drink	18.7	18.4–19.0	12,980
Unflavored	1.0	0.9–1.1	715
Other	17.3	17.0–17.6	12,006
*Single flavor used most often at e-cigarette use initiation*			
Tobacco	7.7	7.5–7.9	5301
Menthol	6.3	6.1–6.5	4382
Mint/wintergreen	1.9	1.8–2.0	1306
Fruit	48.5	48.1–48.9	33,574
Dessert/pastry/bakery	25.8	25.5–26.1	17,872
Candy/chocolate/sweet	4.1	39.5–42.5	2823
Spice	1.0	0.9–1.1	726
Coffee	2.3	2.2–2.4	1570
Alcohol/cocktail	0.3	0.3–0.3	220
Non-alcoholic/non-coffee drink	1.1	1.0–1.2	779
Unflavored	0.1	0.1–0.1	89
Other	0.9	0.8–1.0	591

*Multiple responses were allowed

Table 3 presents the e-cigarette equipment and flavors used by the participants at the time of survey participation. E-cigarette use initiation was reported at 4 years (IQR: 2–6 years) before survey participation, while regular use was reported for 3 years (IQR: 2–5 years). The patterns of equipment use were for the most part similar to the data at e-cigarette use initiation. Even more participants were using advanced devices (variable voltage/wattage) at the time of survey participation. Use of disposable or rechargeable first-generation devices was rare. By far, the most popular nicotine concentration was 1–6 mg/mL (more than twice the use rate at e-cigarette use initiation), which is compatible with the well-documented gradual transition to lower nicotine concentration over time. The most popular flavors were again fruit and dessert/pastry/bakery. Use of tobacco flavors was by far less prevalent compared to e-cigarette use initiation, both as a flavor used regularly and as a single most often used flavor. Only 2.1% of participants reported that the single most often used flavor at the time of survey participation was a tobacco flavor, compared to 7.7% at use initiation. Many participants reported using multiple flavors within the same day.

**Table 3:** E-cigarette equipment and flavor use at the time of the survey (*n* = 69,233)

	%	95% CI	*N*
*Device used*			
Disposable	0.2	0.2–0.2	155
Rechargeable ciga-like with prefilled cartridges	3.1	3.0–3.2	2179
eGo-style	3.5	3.4–3.6	2409
Pod mod	3.0	2.9–3.1	2059
Mechanical device	10.7	10.5–10.9	7388
Variable voltage/wattage (advanced personal vaporizer)	76.7	76.4–77.0	53,128
Something else	1.5	1.4–1.6	1011
*Nicotine concentration*			
0 mg/mL	6.2	6.0–6.4	4258
1–6 mg/mL	82.4	82.1–82.7	57,057
7–12 mg/mL	4.7	4.5–4.9	3238
13–17 mg/mL	0.8	0.7–0.9	561
18–24 mg/mL	1.7	1.6–1.8	1172
25–49 mg/mL	1.3	1.2–1.4	928
50 mg/mL or more	1.1	1.0–1.2	762
Flavor choices (used regularly)*			
Tobacco	7.8	7.6–8.0	5395
Menthol	13.3	13.0–13.6	9217
Mint/wintergreen	9.6	9.4–9.8	6616
Fruit	83.0	82.7–83.3	57,447
Dessert/pastry/bakery	70.5	70.2–70.8	48,823
Candy/chocolate/sweet	46.3	45.9–46.7	32,064
Spice	9.2	9.0–9.4	6394
Coffee	19.3	19.0–19.6	13,385
Alcohol/cocktail	6.9	6.7–7.1	4746
Non-alcoholic/non-coffee drink	13.5	13.2–13.8	9368
Unflavored	0.9	0.8–1.0	630
Other	11.5	11.2–11.7	7945
*Single flavor used most often*			
Tobacco	2.1	2.0–2.2	1481
Menthol	2.5	2.4–2.6	1734
Mint/wintergreen	1.2	1.1–1.3	862
Fruit	49.0	48.6–49.4	33,893
Dessert/pastry/bakery	35.3	34.9–35.7	24,436
Candy/chocolate/sweet	4.4	4.2–4.6	3062
Spice	0.6	0.5–0.7	389
Coffee	1.3	1.2–1.4	903
Alcohol/cocktail	0.3	0.3–0.3	206
Non-alcoholic/non-coffee drink	0.8	0.7–0.9	552
Unflavored	0.2	0.2–0.2	131
Other	1.0	0.9–1.1	680
*Frequency of using different flavors*			
Use multiple flavors in the same day	42.0	41.6–42.4	29,112
Change flavors every 2–3 days	21.1	20.8–21.4	14,574
Change flavors every 4–5 days	6.7	6.5–6.9	4642
Change flavors every week	9.4	9.2–9.6	6474
Change flavors every 2 weeks	8.2	8.0–8.4	5701
Change flavors every month	11.3	11.1–11.5	7826

*Multiple responses were allowed

### Flavors and device choice according to the smoking status

**Fig. 2:**
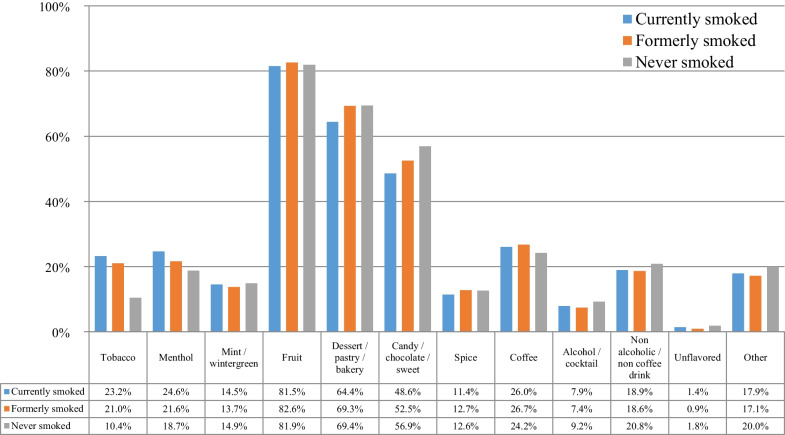
Flavor choice at e-cigarette use initiation among participants who currently smoked, formerly smoked, and never smoked (*n* = 69,233)

Figure 2 presents the choice of flavors at e-cigarette use initiation according to the smoking status at the time of survey participation. For all groups, fruit flavors were the most popular, followed by dessert/pastry/bakery and candy/chocolate/sweet flavors. Statistically significant differences were found for all flavors besides mint-wintergreen. Never smoking participants were more likely to initiate e-cigarette use with fruit and candy/chocolate/sweet compared to those currently and formerly smoking, but the differences were small. For fruit flavors, no statistically significant difference was found between those who never and formerly smoked. Tobacco flavors were more prevalent among those currently smoked compared to those formerly and never smoked, and were least prevalent among those who never smoked (less than half the rate of those currently and formerly smoking).

**Fig. 3:**
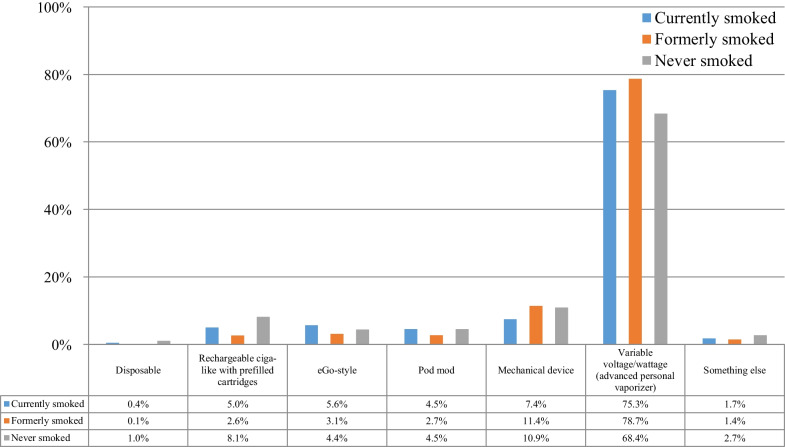
Device choice at the time of survey participation according to the smoking status of participants (*n* = 69,233)

Figure 3 shows the device choice at the time of survey participation according to the smoking status. Small but statistically significant differences were found between groups. Advanced devices were by far the more popular overall, but were statistically less prevalent among those who never compared to those who formerly and currently smoked. Disposables or rechargeable ciga-like devices were rarely used at the time of survey participation.

**Fig. 4:**
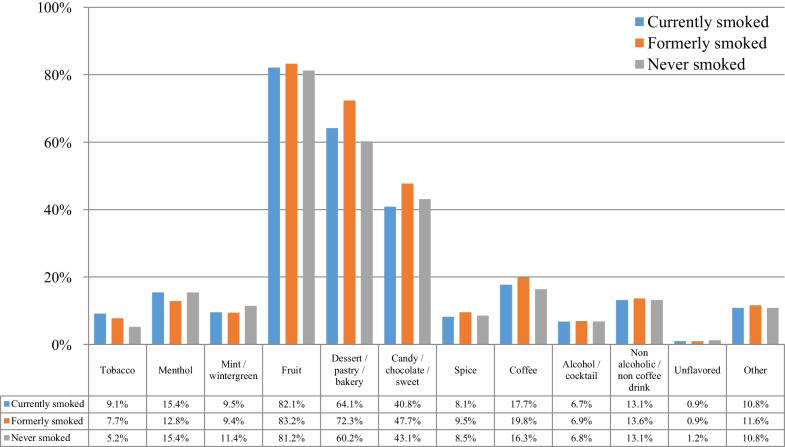
Choice of flavors at the time of survey participation according to the smoking status of participants (*n* = 69,233)

Figure 4 presents the choice of flavors at the time of survey participation according to the smoking status. Again, fruit flavors were the most popular, followed by dessert/pastry/bakery and candy/chocolate/sweet flavors. Tobacco flavors prevalence was substantially lower compared to the period of e-cigarette use initiation for all groups. Dessert/pastry/bakery and candy/chocolate/sweet flavors were more prevalent among those who formerly compared to currently and never smoked.

**Fig. 5:**
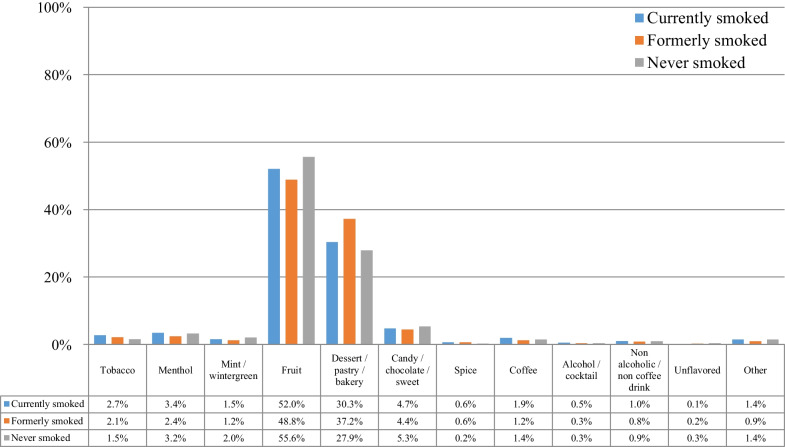
Choice of the one flavor most often used at the time of survey participation according to the smoking status of study participants (*n* = 69,233)

Figure 5 presents the choice of the single most often used flavor at the time of survey participation according to the smoking status at the time of survey participation. Fruit flavors were the most popular, followed by dessert/pastry/bakery. Participants who never smoked were more likely to use fruit flavors compared to those who currently and formerly smoked. However, those who formerly smoked were more likely to use dessert/pastry/bakery compared to the other groups. Minimal use of tobacco flavors was observed in all groups, with those who currently smoked being more likely to use them compared to the other groups.

### Using e-cigarettes at the time of quitting smoking

A sub-analysis of the survey focused on participants who formerly smoked and were using e-cigarettes at the time of smoking cessation. They represented 74.6% of the study sample (*n* = 51,641, Fig. 1).

Table 4 presents the e-cigarette use patterns, equipment, and flavor used by this subgroup of study participants at the time of quitting smoking. From all participants who formerly smoked, 8.3% reported that they were not using e-cigarettes at the time of quitting; they were excluded from the present analysis. The vast majority of this subgroup reported that they would definitely or probably still be smoking today if they had never started using e-cigarettes. Most were using advanced e-cigarette devices, while the most popular nicotine concentration at the time of quitting was 1–6 mg/mL, followed by 18–24 mg/mL. The vast majority considered finding the flavor of preference as an extremely or very important factor in their attempt to quit smoking, and identified fruit, dessert/pastry/bakery and candy/chocolate/sweet as the flavors that were particularly helpful for quitting smoking. The most popular flavor choices at the time of quitting smoking were fruit flavors, followed by dessert/pastry/bakery. Only 15% of participants were using tobacco flavors. Fruit and dessert/pastry/bakery and candy/chocolate/sweet flavors were considered particularly helpful to avoid relapse to smoking, with only 7.3% considering that tobacco flavors could have such a role.

**Table 4:** E-cigarette use patterns, equipment, and flavor use at the time of quitting smoking by participants who formerly smoked (*n* = 51,641)

	%	95% CI	*n*
*At the time of quitting smoking, were you using e-cigarettes: (1)*			
Every day	85.1	84.8–85.4	47,933
Some days	6.6	6.4–6.8	3708
Not at all (2)	8.3	8.1–8.5	4659
*If you had never started using e-cigarettes, would you still be smoking today?*			
Definitely yes	72.2	71.8–72.6	37,265
Probably yes	23.9	23.5–24.3	12,359
Probably no	1.7	1.6–1.8	881
Definitely no	2.2	2.1–2.3	1136
*Device used at time of quitting smoking*			
Disposable	1.9	1.8–2.0	1005
Rechargeable ciga-like with prefilled cartridges	9.8	9.5–10.1	5044
eGo-style	26.8	26.4–27.2	13,827
Pod mod	1.2	1.1–1.3	625
Mechanical device	5.2	5.0–5.4	2666
Variable voltage/wattage (advanced personal vaporizer)	54.4	54.0–54.8	28,081
Something else	0.8	0.7–0.9	393
*Nicotine concentration at time of quitting smoking*			
0 mg/mL	1.5	1.4–1.6	798
1–6 mg/mL	46.9	46.5–47.3	24,220
7–12 mg/mL	19.3	19.0–19.6	9973
13–17 mg/mL	4.2	4.0–4.4	2161
18–24 mg/mL	23.1	22.7–23.5	11,927
25–49 mg/mL	2.7	2.6–2.8	1418
50 mg/mL or more	0.7	0.6–0.8	365
*How important was finding an e-cigarette/e-liquid flavor you liked in your attempt to quit smoking?*			
Extremely important	69.7	69.3–70.1	35,979
Very important	17.6	17.3–17.9	9070
Important	8.7	8.5–8.9	4508
Slightly important	3.1	3.0–3.2	1579
Not important	1.0	0.9–1.1	505
Flavor choices (used regularly) at time of quitting smoking*			
Tobacco	15.0	14.7–15.3	7763
Menthol	18.2	17.9–18.5	9394
Mint/wintergreen	11.5	11.2–11.8	5934
Fruit	83.3	83.0–83.6	43,012
Dessert/pastry/bakery	68.0	67.6–68.4	35,106
Candy/chocolate/sweet	44.5	44.1–44.9	22,986
Spice	9.6	9.3–9.9	4951
Coffee	19.9	19.6–20.2	10,298
Alcohol/cocktail	5.9	5.7–6.1	3050
Non-alcoholic/non-coffee drink	13.1	12.8–13.4	6766
Unflavored	0.7	0.6–0.8	349
Other	9.2	9.0–9.4	4761
*Single flavor used most often at time of quitting smoking*			
Tobacco	5.1	4.9–5.3	2617
Menthol	4.7	4.5–4.9	2430
Mint/wintergreen	1.6	1.5–1.7	841
Fruit	49.3	48.9–49.7	25,483
Dessert/pastry/bakery	30.3	29.9–30.7	15,657
Candy/chocolate/sweet	4.1	3.9–4.3	2105
Spice	1.1	1.0–1.2	593
Coffee	1.8	1.7–1.9	953
Alcohol/cocktail	0.3	0.3–0.3	142
Non-alcoholic/non-coffee drink	0.9	0.8–1.0	476
Unflavored	0.1	0.1–0.1	40
Other	0.6	0.5–0.7	304
*Flavor choices that were particularly helpful for quitting smoking*			
Tobacco	9.3	9.0–9.6	4813
Menthol	11.7	11.4–12.0	6020
Mint/wintergreen	7.4	7.2–7.6	3820
Fruit	60.8	60.4–61.2	31,393
Dessert/pastry/bakery	48.9	48.5–49.3	25,277
Candy/chocolate/sweet	29.7	29.3–30.1	15,327
Spice	7.1	6.9–7.3	3649
Coffee	13.9	13.6–14.2	7201
Alcohol/cocktail	4.7	4.5–4.9	2438
Non-alcoholic/non-coffee drink	9.6	9.3–9.9	4943
Unflavored	0.5	0.4–0.6	264
Other	8.0	7.8–8.3	4130
*Flavor choices that were particularly helpful to avoid relapse to smoking*			
Tobacco	7.3	7.1–7.5	3792
Menthol	11.5	11.2–11.8	5925
Mint/wintergreen	8.8	8.6–9.0	4525
Fruit	72.1	71.7–72.5	37,244
Dessert/pastry/bakery	61.9	61.5–62.3	31,958
Candy/chocolate/sweet	40.6	40.2–41.0	20,981
Spice	9.2	9.0–9.4	4735
Coffee	19.1	18.8–19.4	9857
Alcohol/cocktail	6.6	6.4–6.8	3425
Non-alcoholic/non-coffee drink	12.9	12.6–13.2	6641
Unflavored	0.7	0.6–0.8	363
Other	9.3	9.0–9.6	4816

(1) Data on participants who were using e-cigarettes every day or on some days when quitting smoking are presented in the rest of the table

(2) These participants were excluded from the rest of the analysis in the present table

*Multiple responses were allowed

**Fig. 6:**
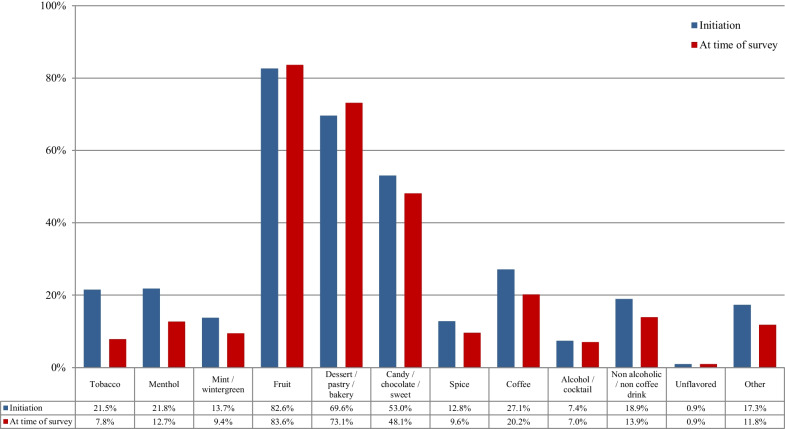
Transition in flavor choice from e-cigarette use initiation to the time of survey participation by participants who formerly smoked and were using e-cigarettes at the time of quitting smoking (*n* = 51,641)

Figure 6 presents the transition in flavor choice from e-cigarette use initiation to the time of survey participation by participants who formerly smoked and were using e-cigarettes at the time of quitting smoking. A small increase in prevalence of fruit and dessert/pastry/bakery use was observed over time, which were the most popular choices. A substantial decrease in the use of tobacco flavors to almost 1/3rd the use rate at e-cigarette use initiation was also reported.

**Fig. 7:**
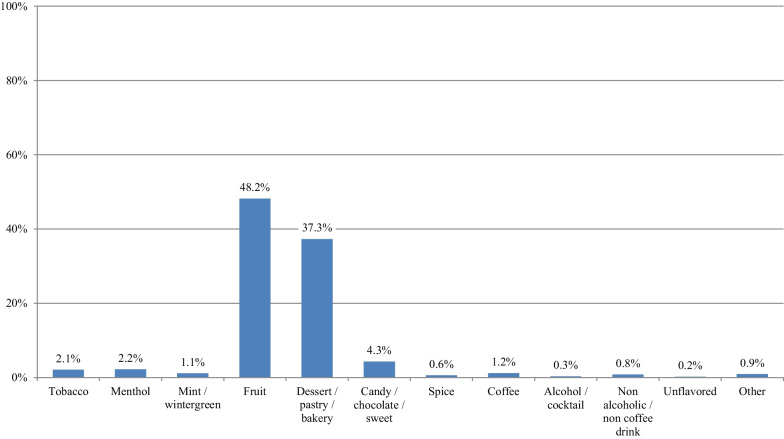
Single most often used flavor at the time of survey participation by participants who formerly smoked and were using e-cigarettes at the time of quitting smoking (*n* = 51,641)

Figure 7 shows the single most often used flavor at the time of survey participation by participants who formerly smoked who were using e-cigarettes at the time of quitting smoking. Again, fruit flavors were the most popular, followed by dessert/pastry/bakery. Use of tobacco flavors was rare.

## Discussion

This study represents the largest survey ever performed on e-cigarette use in terms of sample size, with almost 70,000 participants. The main findings were that non-tobacco flavors, especially fruit and dessert/pastry/bakery flavors, were the most prevalent choices of the established, dedicated adult US vapers who participated in this study. These flavors were particularly popular not only during long-term e-cigarette use, but also at the period of e-cigarette use initiation. Additionally, they were popular at the time of smoking cessation among those who formerly smoked. Fruit and dessert/pastry/bakery flavors were also considered particularly important in their effort to quit smoking and to prevent relapse to smoking. Tobacco flavors were generally used by a minority of the study participants, and their use prevalence decreased substantially over time.

The patterns of e-cigarette flavor use observed herein are in agreement with a cross-sectional study examining the responses of more than 20,000 participants from the USA [15]. Additionally, a recent longitudinal study examined changes in flavor use patterns in long-term vapers over a period of 5 years and found a transition to sweet flavors with a significant reduction in the use of tobacco, menthol, and mint flavors [16]. Importantly, almost all participants in that study were using more than one flavor on a regular basis, while only 11.2% reported that tobacco was their preferred flavor. Multiple flavor use has been observed since the early days of e-cigarettes [8]. This may be related to olfactory fatigue that is observed when the same flavor is used for prolonged time [8, 17] or due to a significant decrease in pleasantness with time even when the odor is initially pleasant [18].

Limited data exist on the issue of flavor choice by people who formerly smoked and used e-cigarettes to quit. In this study, we examined the patterns of flavor use at the time of smoking cessation in this subgroup. Non-tobacco flavors were the predominant choice at the time of smoking cessation, and they were considered particularly helpful both for quitting smoking and to prevent relapse. A longitudinal study examining factors associated with past 30-day abstinence from cigarette smoking among people buying an e-cigarette found that those who used non-tobacco flavors were 30% more likely to report smoking abstinence compared to those using tobacco flavor [19]. Data from the 2018–2019 Tobacco Use Supplement-Current Population Survey (TUS-CPS) showed that people who smoked and used e-cigarettes with non-tobacco flavors were more likely to make a quit attempt and to successfully quit compared to those exclusively using non-flavored or tobacco-flavored e-cigarettes [20]. An analysis of the waves 1 and 2 of the Population Assessment of Tobacco and Health (PATH) study focusing on young adults reported that vapers of one and multiple non-tobacco/non-menthol flavors were more likely to have reduced or quit smoking over the past year compared to those who did not use e-cigarettes [21]. Another analysis of waves 1 to 4 of the PATH survey found that vaping non-tobacco flavors was no more associated with youth smoking initiation than vaping tobacco flavors but was associated with increased adult smoking cessation [22]. A study following-up 886 participants who reported dual use for 2 years (from 2016 to 2018) observed that the use of fruit and other sweet flavored e-liquids was positively related to a transition away from cigarettes compared to those using tobacco flavors [23]. Finally, a recent systematic review concluded that the availability of a variety of flavors in e-cigarettes might facilitate complete substitution for cigarettes, because it is an important factor in e-cigarette appeal among people who smoke [24].

While our data and the above-mentioned studies raise the possibility that non-tobacco flavors may have a role in smoking cessation, the evidence on the association between flavored e-cigarettes and smoking relapse is even scarcer. Herein, we found that the vast majority of participants considered fruit and dessert/pastry/bakery and candy/chocolate/sweet flavors as important to avoid relapse. It is unclear whether this represents a way to be distracted from the tobacco flavor in order to reduce smoking craving, or that they just do not need the tobacco flavors any more but feel the desire to experiment with new flavors [8]. It may also be the result of an aversion to the flavor of tobacco over time or an improvement in taste function after smoking cessation, with the latter being verified in the clinical setting [25–27]. Interestingly, while flavors such as candy or fruits were associated with greater rates of enjoyment compared to tobacco flavors and more satisfaction compared to smoking, those using tobacco flavors were more likely than those using candy or unflavored products to vape in order to avoid relapse to smoking [28]. However, this study was performed in people who had quit smoking recently (in the last 2 years), and it is unclear whether the findings could be applicable to longer-term former-smoking people as those in our study sample. Understanding the effect of flavors on smoking relapse is expected to be complex due to the observed transition in flavor use over time, poly-flavor use and perhaps interindividual characteristics in personal preference that may affect flavor choices.

Regulatory concerns over e-cigarette flavors emerge from evidence that youth may be more likely to use non-tobacco flavors [29, 30], although it is unclear if this is linked to subsequent smoking uptake [22, 30]. However, any regulation on e-cigarette flavors should consider the balance between protecting from unintended use by some population subgroups (e.g., by adolescents or people who have never smoked) and avoiding adverse effects and potential harm to other subgroups (e.g., by preventing people who smoke from switching to e-cigarettes in a harm reduction approach to quitting smoking). Our data raise the possibility that an overly restrictive regulation, such as banning the sales of specific flavor groups (particularly fruit and dessert/pastry/bakery flavors), might have unintended consequences, preventing people who smoke from switching to e-cigarette use and/or increasing the relapse rate among those who formerly smoked and have managed to quit with the help of e-cigarettes. Due to the harm reduction potential and possible health benefits of switching from smoking to e-cigarette use, any regulatory decisions will be much more complex compared to similar past decisions on the ban of flavors in tobacco cigarettes. Therefore, more evidence is needed in order to clarify the role of flavors in smoking cessation and relapse prevention, and regulators should be careful in striking the right balance in order to avoid unintended adverse public health effects.

A major limitation of the study is the cross-sectional design and the recruitment of a convenience sample of dedicated vapers. The sample was not representative of the general US adult population, and the study was not designed or intended to estimate the population prevalence or frequency of e-cigarette flavor use. The flavor preferences and patterns of e-cigarette use reported by the present sample of dedicated vapers may more closely represent those who exclusively use e-cigarettes on a daily basis rather than the majority who experiment or use e-cigarettes occasionally. Still, this survey presents the patterns of use of a very large sample of adult US vapers, most of which self-reported that they were successful in quitting smoking with the help of e-cigarettes. While flavors seem to play an important role in their smoking cessation attempt, it should be mentioned that other characteristics, such as the more prevalent use of advanced e-cigarette devices compared to ciga-likes or pod systems, may also contribute to a successful quit attempt. Therefore, future studies should include more questions specifically addressing the trajectory of smoking habits had flavors not been available at the time of making a quit attempt. Major developments in e-cigarette products, such as the marketing of nicotine salts and higher nicotine concentrations were not covered by this study. Additionally, participants were asked about past flavor choices, which may introduce some recall bias. Finally, the findings in this study are not necessarily applicable to vapers in Europe or other regions, and more studies are needed to examine the patterns of flavor use in different populations.

In conclusion, this cross-sectional study of a very large sample of US adults using e-cigarettes, most of whom were formerly smoking, identified the importance of non-tobacco flavors in e-cigarette use initiation and sustained use, and their potential contribution to smoking cessation and relapse prevention. This information should be considered by regulators in order to avoid unintentional adverse effects of over-restrictive regulation on e-cigarette flavors.

### Supplementary Information

**Additional file 1. Table 1.** Residence state of study participants (n = 69,233)
