# Supplementary material for: Patterns of flavored e-cigarette use among adult vapers in the USA: an online cross-sectional survey of 69,233 participants
Source: Harm Reduct J. 2023 Oct 14;20:147. doi: 10.1186/s12954-023-00876-w (PMC10576309; doi:10.1186/s12954-023-00876-w)
Supplement: Supplementary file 1 — Additional file 1. Table 1. Residence state of study participants (n = 69,233) [file 12954_2023_876_MOESM1_ESM.docx]

**Supplementary table 1.** Residence state of all participants (n = 69,233).

| **States** | **% (n)** |
| --- | --- |
| Alabama | 2.4% (1630) |
| Alaska | 0.2% (165) |
| Arizona | 2.4% (1649) |
| Arkansas | 1.2% (830) |
| California | 6.4% (4427) |
| Colorado | 1.7% (1176) |
| Connecticut | 1.0% (665) |
| Delaware | 0.3% (222) |
| District of Columbia | 0.1% (37) |
| Florida | 5.6% (3876) |
| Georgia | 3.8% (2651) |
| Hawaii | 0.4% (255) |
| Idaho | 0.7% (486) |
| Illinois | 4.0% (2798) |
| Indiana | 2.9% (2042) |
| Iowa | 1.4% (994) |
| Kansas | 0.9% (638) |
| Kentucky | 2.6% (1811) |
| Louisiana | 1.2% (843) |
| Maine | 0.4% (286) |
| Maryland | 1.9% (1306) |
| Massachusetts | 1.7% (1144) |
| Michigan | 3.1% (2151) |
| Minnesota | 1.3% (886) |
| Mississippi | 1.0% (717) |
| Missouri | 2.2% (1494) |
| Montana | 0.3% (174) |
| Nebraska | 0.7% (453) |
| Nevada | 1.0% (714) |
| New Hampshire | 0.6% (428) |
| New Jersey | 2.0% (1379) |
| New Mexico | 0.5% (320) |
| New York | 4.5% (3120) |
| North Carolina | 3.0% (2059) |
| North Dakota | 0.2% (169) |
| Ohio | 5.5% (3823) |
| Oklahoma | 1.8% (1228) |
| Oregon | 1.1% (773) |
| Pennsylvania | 4.5% (3085) |
| Rhode Island | 0.3% (218) |
| South Carolina | 1.6% (1104) |
| South Dakota | 0.3% (193) |
| Tennessee | 4.5% (3082) |
| Texas | 6.7% (4663) |
| Utah | 1.1% (732) |
| Vermont | 0.2% (145) |
| Virginia | 2.9% (2032) |
| Washington | 2.1% (1452) |
| West Virginia | 1.1% (763) |
| Wisconsin | 1.8% (1221) |
| Wyoming | 0.3% (218) |
| Missing (no response) | 0.7% (506) |
